# Implementation and effectiveness of an interprofessional educational intervention on patient safety in perinatal care: a multi-method, prospective evaluation study

**DOI:** 10.1186/s12909-026-09907-z

**Published:** 2026-07-09

**Authors:** Katharina Averdunk, Nikoloz Gambashidze, Angela Klein, Matthias Weigl

**Affiliations:** 1 Institute for Patient Safety (IfPS), Medical Faculty, University Hospital Bonn, Bonn, Germany; 2 Gynaecological Psychosomatics, Clinic for Gynaecology and Gynaecological Oncology, University Hospital Bonn, Bonn, Germany; 3 Institute for Patient Safety (IfPS), University Hospital Bonn, Venusberg-Campus 1, Bonn, 53127 Germany

**Keywords:** Interprofessional education, Patient safety, Implementation, Interprofessional collaboration, Perinatal care, Obstetrics

## Abstract

**Background:**

Interprofessional collaboration is fundamental to high-quality and safe care – particularly in perinatal care. Interprofessional education (IPE) for students in healthcare professions has significant potential for enhancing interprofessional competencies and patient safety. However, current evidence on effectiveness of IPE is limited, particularly due to various methodological constraints in evaluation studies such as a lack of prospective data, structured outcome measures, and student involvement.

**Methods:**

In our project *SiGerinn*, students from nursing, midwifery, and medicine received IPE on patient safety. We employed a prospective, multi-method evaluation study over a two-year implementation period. Structured surveys on self-reported competency levels among program participants were collected at baseline and follow-up, complemented by in-depth interviews with stakeholders to assess the project’s implementation status.

**Results:**

Prospective statistical analyses revealed highly significant improvements in competency levels for interprofessional collaboration, communication with patients and relatives, and interprofessional communication techniques. Competency gains varied across student groups, being highest among midwifery students. Moreover, survey and interview statements revealed strong acceptance and perceived benefits from the IPE patient safety intervention. Interview findings further emphasised the uptake of a disseminating role among participating students by peer knowledge sharing.

**Conclusion:**

Our results show positive effects on skill development and encouragement through an IPE intervention on patient safety in perinatal care. Despite several methodological limitations such as small sample sizes, consensus-based outcome measures, and self-reported skill level assessments, our findings provide a starting point for future IPE attempts on patient safety education in clinical settings. Furthermore, obtained insights suggest the value of integrating IPE into health professionals’ curricula to initiate sustainable learning.

**Supplementary Information:**

The online version contains supplementary material available at 10.1186/s12909-026-09907-z.

## Background

Interprofessional (IP) collaboration is crucial to ensure high-quality and safe care [1–3]. Conversely, conflicts and ineffective teamwork can lead to insufficient patient safety [4]. In perinatal care, particular challenges exist for IP collaboration with respect to shared power, differing perspectives on care practices, communication barriers, and ‘professional stereotyping’ as a result of separated educational programs [4, 5]. To overcome these challenges successfully, the need for developing IP competencies at early stages of training and education has been repeatedly emphasised [4, 6–10]. Nevertheless, interprofessional education (IPE) for students in perinatal care have been rarely implemented.

IPE and teamwork interventions can significantly enhance IP competency development and, in turn, improve patient safety [4, 7, 10–15]. However, there is a significant lack of evidence on how to design suitable educational interventions, and effectively implement and evaluate IPE interventions in clinical practice, particularly in perinatal care [11]. Hence, robust data on IPE effectiveness is limited, majorly due to lack of evaluations in real-world clinical care as well as significant methodological shortcomings, for example, a lack of longitudinal measures [11, 13, 16–18]. Specifically, despite the recent growth in the availability of IPE for medical and midwifery students in German-speaking countries [19], evidence supporting its effectiveness in competency development is missing. Overall, students are seldom engaged in evaluation studies examining outcomes of patient safety and IP collaboration [10, 20]. Concurrently, previous research has shown that implementing IPE is impeded by significant barriers such as conflicting schedules across professions, varying interests, and strong dependence on clinical leaders’ support [4, 9, 21–23]. Consequently, there is an urgent need for IPE interventions for students, particularly in high-risk care settings such as perinatal care, with reliable evidence on effectiveness and implementation of IPE. We therefore established the IPE project *SiGerinn* (Safety and Interprofessionality in Perinatal Care – Together and from the Beginning, German: *Sicherheit und Interprofessionalität in der Geburtshilfe – Gemeinsam und von Beginn an*). This IPE intervention comprised practice-focused, interprofessional patient safety education for medical, midwifery and nursing students in a postpartum care ward in a German university hospital.

Considering the aforementioned constraints on reported IPE interventions in perinatal care, our evaluation study sought to evaluate both effectiveness of the intervention in terms of IP competency development, and its implementation status. Effectiveness of IPE programs is typically evaluated using either primary or secondary (intermediate) outcomes [7, 18]. In patient safety studies, primary outcomes include, for example, adverse events [24–26] or specific clinical outcome sets [4, 12]. Secondary outcomes capture general IP competencies such as communication and attitude development [10, 11, 14, 20, 27–29], patient satisfaction [15, 30] or perceived relevance of the course [14, 20, 31]. Recent publications evaluating IPE programs also measured combinations of outcomes [15, 28, 30]. Altogether, studies on insights into the actual implementation status of IPE and related processes in perinatal care settings are rare [19, 20, 22].

To the best of our knowledge, we report for the first time an IPE intervention on patient safety for the three key student groups in perinatal care, i.e., students from medicine, nursing, and midwifery study programs. In consideration of didactic principles and a structured effect evaluation in alignment with pre-defined learning outcomes, our mixed-method evaluation approach provides valuable contributions to the IPE community and shall inform similar initiatives - in and beyond perinatal care.

### Objective

Our prospective evaluation study aimed to assess the effectiveness of a newly designed IPE intervention on patient safety in promoting students’ interprofessional competencies (objective 1), and to evaluate the implementation status after two years of providing IPE on patient safety (objective 2).

## Methods

### Development and implementation of the IPE intervention *(SiGerinn)*

Within a stepwise procedure, we developed an IPE intervention on patient safety for students from medicine, midwifery, and nursing study programs. Psychologists, physicians, nurses and midwives were involved throughout the development process and project implementation. The program aimed to foster IP competencies among students to support safety-focused, collaborative care practices. Program contents were informed by the Canadian National Interprofessional Competency Framework (CNICF), that emphasises the combined development of essential knowledge, skills, and attitudes for IP collaboration [32]. By integrating these domains, our educational content comprised various methodical and behavioural components, addressing the need for holistic approaches in perinatal care. Based on prior review of the literature base on IP competencies, collaboration, and communication in and beyond perinatal care [4, 22, 33–36], we selected four key competencies for our IP patient safety intervention: IP communication, patient-centredness, role clarification, and IP conflict resolution. For each, we defined a competency statement and aligned learning objectives according to CHICF, respectively [32]; cf., Appendix I, Table 3. Further development of didactic methods as well as the effect assessment was intended to be constructively aligned with these learning objectives [37].

During implementation, *SiGerinn* was part of an ‘interprofessional training ward’ (IPSTA) program in a postpartum care ward within a university hospital in Germany. Its obstetric department has around 3,000 deliveries per annum. The IPSTA program provided a structured curriculum including collaborative work on authentic patient cases, sharing feedback, and the development of an interprofessional identity. The duration was scheduled to be three weeks in 2023 and two weeks in 2024. Participants included students from medicine, midwifery, and nursing study programs. IPSTA program participation was limited to one student from each profession per cycle, respectively, to ensure individualised mentoring as well as frequent opportunities to collaborate on patient’s bed-side. While medical students could apply for participation as part of their postpartum practical training, midwifery and nursing students were selected and invited. Our IPE intervention on patient safety was a theoretical teaching unit within the IPSTA program and consisted of two one-hour learning sessions and one reflection session. In alignment with our pre-defined key competencies, i.e., IP communication, patient-centredness, role clarification, and IP conflict resolution, educational contents comprised principles of IP collaboration in the context of patient safety, with a particular focus on challenges encountered in care practice. Applied didactics emphasised participatory learning, with a strong focus on engaging students to interact and enabling interprofessional reflective case work. Instructors were a nurse or a midwife, with clinical experience as well as academic background in patient safety. Details on educational content and procedures are described elsewhere [23] or are available upon request by the corresponding author.

### Prospective, multi-method evaluation study of the IPE intervention *(SiGerinn)*

#### Design

We established a within-subject, pre-post study design with a multi-methods approach comprising quantitative surveys with program participants as well as semi-structured in-depth interviews with key stakeholders. We used SQUIRE-EDU guideline for reporting of our results [38].

#### Participants and sampling

All program participants (i.e., students from medicine, nursing, and midwifery study programs) were eligible for evaluation survey. After the project period, all participants received an invitation and a reminder to participate the interview study. Besides student participants, interviews also included project partners (i.e., health professionals who worked closely with participants in care practice or contributed to interprofessional learning sessions). In alignment with recommendations [39], we aimed to conduct a minimum of five individual interviews.

#### Data collection

Student participants completed identical questionnaires before the first learning session (baseline) and approximately three weeks later during the reflection session (follow-up). Using participant codes, we assessed individual competency levels at two time points, allowing for identification of potential changes over time [40]. Stakeholder interviews took place in-person in the local department of gynaecology, or via video call.

#### Measures

To measure the specific contents and competencies being addressed within our intervention, we used a combination of self-developed and established survey metrics.

Prior to baseline, the study team established a consensus on content- and context-specific evaluation criteria for expected IP competency improvements over time. Applying an outcome-based evaluation approach, assessment criteria were constructively aligned with pre-defined learning objectives [37, 40]. These objectives and related educational contents were phrased and iteratively refined as survey items. Face validity of these items was tested with professionals being involved in the IPE intervention and on-site teaching. Using a cognitive pre-test, we additionally piloted the survey items with local care professionals. Eventually, we composed all statements into three pre-defined survey categories for standardised self-assessment of IP competency levels (six-point Likert scale: 1 = low, 6 = high); for complete questionnaire, cf., Appendix II, Table 4. Our three survey categories on competency development were:

- (1) Interprofessional collaboration (7 items): This category captured competencies and skills on IP communication, mutual support, and professional roles. Internal consistency was assessed with Cronbach’s Alpha (CA) = 0.88.
- (2) Communication with patients and relatives (6 items): Items measured communication competencies with patients and relatives as well as involving and encouraging them for patient safety behaviours (CA = 0.63).
- (3) Interprofessional communication techniques (9 items): This category comprised items for specific patient safety behaviours within the care team (e.g., Speak Up, standardised patient handover, Briefing and Debriefing; CA = 0.89).

Additionally, we aimed to assess students’ perceived value of IPE experiences. We therefore included category (4) Perceptions of interprofessional education. We utilised three items from the German Interprofessional Attitudes Scale [41], a well-established and validated survey tool that has been previously applied in German-speaking healthcare and teaching settings. In particular, we assessed participants appraisals concerning the perceived value of interprofessional education in the course of practice training (rated on a five-point Likert scale: 1 = disagree, 5 = agree; CA = 0.82).

The interview guide for the semi-structured, in-depth interviews aligned with the implementation outcomes of interest. Consistent with Proctor et al.’s taxonomy [42], questions addressed acceptability, adoption, appropriateness, feasibility, fidelity, penetration, and sustainability of the intervention (for survey instruments, cf., Appendix II, Table 5).

#### Data analysis

For within-subject survey data, we computed descriptive and inferential statistical analyses. Using analyses of variances, we tested for potential baseline differences between student groups. For the overall study group, we tested for prospective changes over time with paired t-tests, i.e., longitudinal comparison of reported IP competency levels. Additionally, difference tests over time were computed for each student group, respectively. To account for multiple testing, we adjusted significance levels using Bonferroni correcting procedure (*p* = 0.05/ 12 tests, i.e., 3 professional groups * 4 survey outcomes, resulting in an adjusted *p* = 0.004). For comprehensiveness of reporting, we determined changes for individual competencies (single items) over time. All statistical analyses were computed with IBM SPSS (29.0 Chicago, IL, US). Interview data was transcribed automatically using R (4.3.2 (2023-10-31) Rstudio, Inc.). After checking the transcripts for correctness, we conducted thematic content analysis to evaluate implementation factors, considering predefined implementation outcomes.

#### Ethical considerations

Our evaluation study received ethical approval by the Ethics Committee of the Medical Faculty, University of Bonn, Germany (#203/22). Study participation was voluntary. Study participants received detailed information on privacy, data processing, and data utilisation. All participants provided informed consent for study participation.

## Results

### Objective 1: evaluating IP competency development over time – survey results

We conducted nine program cycles between June 2023 and December 2024, including a total number of 24 participants. Two to three students of different professional groups participated in each learning session (mean = 2.67). Eventually, 20 participants (87%) answered pre and post questionnaires completely. The remaining four participants answered only one questionnaire due to partial absence in the learning sessions due to illness. Since a considerable amount of learning contents were missing, we did not invite these students to participate in the survey study yet, so that these cases were excluded from further statistical analyses. Our final sample (*n* = 20) consisted of four medical students (mean number of completed semesters = 11.75 (SD = 0.96); range 11–13), nine midwifery students (mean semesters = 3.33 (SD = 1.0); range 2–5), and seven nursing students (mean semesters = 5.14 (SD = 0.38); range 5–6).

Concerning potential differences at baseline, we observed no significant differences in competency ratings and for perceptions of interprofessional education between professions (Table 1).

**Table 1:** Group differences in self-perceived competency levels at baseline

Survey category	Student group			Test for between group difference (ANOVA)	
	Nursing	Midwifery	Medicine		
	M (SD)	M (SD)	M (SD)	F (df=19)	p
Interprofessional collaboration	4.53 (0.57)	4.38 (0.57)	4.07 (0.85)	0.68	0.52
Communication with patients and relatives	3.76 (0.87)	3.72 (0.50)	3.96 (0.53)	0.18	0.83
Interprofessional communication techniques	3.37 (0.83)	3.12 (0.65)	3.69 (0.74)	0.86	0.44
Perceptions of interprofessional education	4.90 (0.25)	4.96 (0.11)	5.00 (0.00)	0.46	0.64

*M* Mean, *SD* Standard deviation, *p* Significance level

**Fig. 1:**
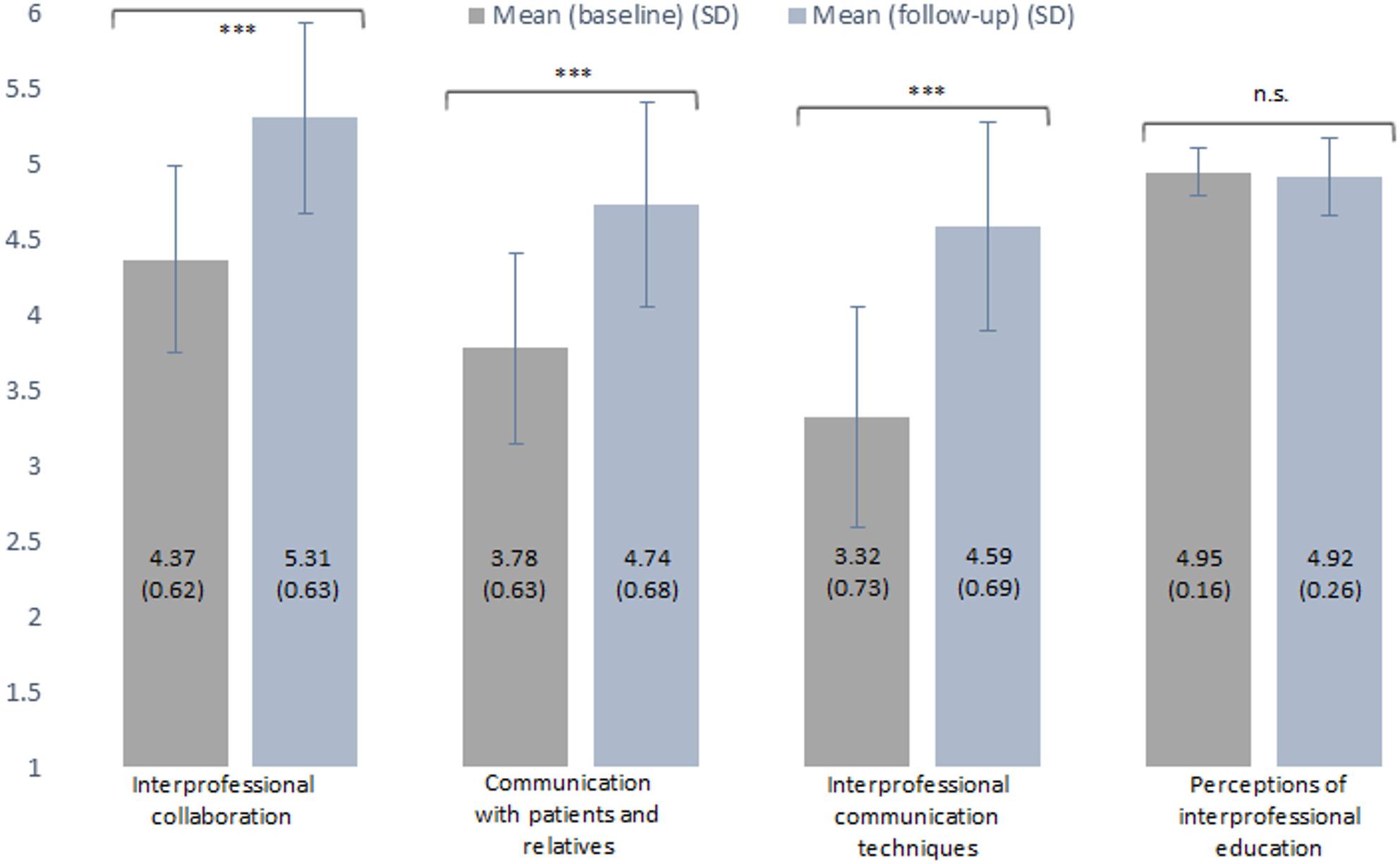
Self-perceived competency levels for baseline and follow-up (total sample). Notes: *M* Mean, *SD* Standard deviation, *p* Significance level: *p* < 0.001 (***), *n.s.* not significant

For the total sample, we identified significant mean differences with large estimated effect sizes (Hedges’ g ≥ 0.8) concerning participants’ competency development over time (Fig. 1). For interprofessional collaboration: M(baseline) = 4.37 (SD = 0.62), M(follow-up) = 5.31 (SD = 0.63), t (19) = 6.6, *p* < 0.001, g = 1.42. For communication with patients and relatives: M(baseline) = 3.78 (SD = 0.63), M(follow-up) = 4.74 (SD = 0.68), t (19) = 6.31, *p* < 0.001, g = 1.35. For interprofessional communication techniques: M(baseline) = 3.32 (SD = 0.73), M(follow-up) = 4.59 (SD = 0.69), t (19) = 6.76, *p* < 0.001, g = 1.45. No significant change over time was observed in perceived relevance of interprofessional education: M(baseline) = 4.95 (SD = 0.16), M(follow-up) = 4.92 (SD = 0.26), t (19) = 0.81, *p* = 0.428, g = 0.17.

Within additional analyses, we tested for reported competency changes over time for each student group, respectively (Table 2). After adjusting for significance levels, we identified significant mean differences and large estimated effect sizes for all of the three competency categories for midwifery students. For nursing students, we observed significant changes in two competency categories (Interprofessional collaboration and Interprofessional communication techniques). For medical students, we did not obtain significant changes, although results showed a positive trend for the three competency categories.

**Table 2:** Self-perceived competency levels per student group (baseline and follow-up) with test for differences

Survey category		Pre-Post Evaluation		Test for differences		
	Student group	Baseline	Follow-up			
		M (SD)	M (SD)	t	p	g
Interprofessional collaboration	Nursing	4.53 (0.57)	5.33 (0.65)	6.41	**< 0.001**	**2.11**
	Midwifery	4.38 (0.57)	5.60 (0.35)	8.79	**0.001**	**2.65**
	Medicine	4.07 (0.85)	4.61 (0.65)	0.92	0.427	0.33
Communication with patients and relatives	Nursing	3.76 (0.87)	4.57 (0.98)	2.58	0.042	**0.85**
	Midwifery	3.72 (0.50)	4.96 (0.54)	6.97	**< 0.001**	**2.10**
	Medicine	3.96 (0.53)	4.54 (0.28)	2.11	0.125	0.76
Interprofessional communication techniques	Nursing	3.37 (0.83)	4.62 (0.91)	5.69	**0.001**	**1.87**
	Midwifery	3.12 (0.65)	4.72 (0.60)	5.16	**< 0.001**	**1.55**
	Medicine	3.69 (0.74)	4.25 (0.44)	1.59	0.209	0.58
Perceptions of interprofessional education	Nursing	4.90 (0.25)	4.86 (0.38)	1.00	0.356	0.33
	Midwifery	4.96 (0.11)	5.00 (0.00)	1.00	0.347	0.30
	Medicine	5.00 (0.00)	4.83 (0.33)	1.00	0.391	0.36

*M* Mean, *SD* Standard deviation, *p *Significance level, bold if *p* (adjusted) ≤ 0.004, *g *Effect size, bold if Hedges’ *g* ≥ 0.8

For comprehensive reporting, we also provide descriptive statistics and test results of all individual items in the appendix (cf., Appendix III, Table 6). We obtained significant changes over time for most competency statements on Interprofessional communication techniques and with varying results for Interprofessional collaboration and Communication with patients and relatives. Specifically, we observed significant changes in items concerning team coordination and role clarity, patient encouragement and participation, and ability to use communication techniques such as Speak Up, Briefing and Debriefing, and Closed Loop Communication.

### Objective 2: interview results on implementation status

We conducted five interviews with two project partners and three students who participated in the IPE intervention (i.e., one from midwifery, two from medicine study programs). Interview duration was between 12 and 18 min (M = 14.8). We classified interview results and key statements (in italics) within the following major categories:

#### Satisfaction

Interviews revealed very high acceptance of the IPE intervention on patient safety. Participants emphasised its practical relevance, involvement of personal experiences, and its feasibility in small-group and interprofessional formats.

Professional (P): *‘I found it very practical*,* very clear*,* very engaging*,* in the discussions. I had the impression that the participants were very involved and were also able to contribute their own experiences or perspectives.’*

Student (S): *‘The format was*,* of course*,* excellent. In the small group*,* you can learn in a much more specific way*,* especially since more people actively participate*,* share their own stories*,* or discuss their own cases. That was absolutely perfect.’*

#### Effects

Stakeholders consistently reported positive effects, especially for perceived encouragement and acquired communication techniques, particularly Speak Up and Closed Loop Communication.

S: *‘I came out of it feeling more confident. I thought*,* ‘We’ve learned this now*,* I’m supposed to say it* [patient safety concerns], *and if I get a dumb comment*,* then that’s how it is.’ But most of the time*,* you don’t even get a dumb comment. I thought that was really good.’*

P: *‘What I really noticed in the reflection sessions was the strengthened self-confidence and self-awareness*,* and also specific situations where they said*,* ‘I applied it there’ or ‘I spoke up there.’ It’s really nice to see that and to hear them say*,* ‘I dared to do it*,* and yes*,* it was difficult or challenging*,* but I’m glad I did it’. We heard that several times.’*

#### Applicability

Participants indicated that the program helped in applying communication techniques in clinical practice. However, they questioned full applicability and transfer of the educational content in care practice due to high workload, time constraints, and staff shortage.

S: *‘What stands out to me from the clinical everyday routine is the Closed Loop Communication* […]. *That stuck with me*,* and I applied it. And also Speak Up* […]. *I’ve tried to bring that up or remind myself of it in certain situations.’*

S: *‘I think*,* it was simply an opportunity where we could actually carry out what we would otherwise have wanted to do.* […] [But] *it’s just utopian to think that we could implement it like this. And*,* that’s sad*,* but honestly*,* I don’t see it working in everyday practice.* […] *Too much workload for one person.* […] *Time and staff shortages.’*

#### Implementation status and process

Project partners highlighted the good match of the patient safety intervention with the IPSTA learning environment, indicating program feasibility with sustainable achievements in terms of sharing expertise in IP teams. In the light of reported structural differences among study curricula as well as the double load of instructors in terms of routine care and individual mentoring, situational flexibility was deemed a critical success factor.

P: *‘I think they* [IPSTA and SiGerinn] *complement each other really well. At the beginning*,* it was definitely a challenge to figure out or get to know each other’s content. What does patient safety bring? What do we bring? What are the key areas? Where can we build on each other? Where do we need to clearly define the boundaries? Because some are really experts in certain areas*,* and where can we use that to integrate it* […]*?’*

P: *‘I think the organisation was often very uncomplicated.* […] *We had to be quite flexible*,* unfortunately*,* since someone was sick or something came up.’*

#### Dissemination

Stakeholders mentioned the limited scope of the program. However, partners emphasised its ‘pioneering’ character, expecting participants to apply and disseminate their acquired skills among peers. Actual uptake of this role was supported by students’ responses.

P: *‘The idea behind this is a so-called lighthouse project*,* where these three participants receive a lot of input*,* attention*,* and guidance.* […] *They then go out into the world*,* preferably staying at the* [local hospital], *as some participants have already done. And then*,* they spread the knowledge and acquired skills. Nevertheless*,* it would be desirable if we could involve more participants.’*

S: *‘In terms of reach*,* I would say the whole* [program] *was relatively limited.* […] *I was the only one* [out of four medical students] *who got to do it. The others would have liked to as well.’*

S: *‘I know that I’ve talked about these things* [Closed Loop Communication, Speak Up] *with other students or friends and shared that with them. I could easily do that with these little*,* specific things*,* and I think they received it well.’*

Moreover, integration of educational contents was notable on an organisational level: First, in terms of outreach into care routines, and second, for its potential ‘preparatory work’ for future projects.

P: *‘Generally*,* things like the ISBARR* [Introduction–Situation–Background–Assessment–Recommendation–Read back] *scheme and other tools are more present.* […] [The ward leader] *always welcomes that very much. I believe there’s still a lot of room for growth*,* but things are definitely moving forward.’*

## Discussion

Acquiring competencies for effective IP collaboration is a key challenge for students in health professions, particularly, those working in high-stakes care environments, such as perinatal care. Given the lack of empirical evidence for the effectiveness of IPE approaches, we introduced a novel program on patient safety and IP collaboration for students in perinatal care. While drawing upon on an established IP competency framework and in constructive alignment with pre-defined learning objectives [32, 37], we developed a tailored didactic approach with context- and content-specific assessment criteria. Our evaluation study aimed to assess competency development over time among students participating in the IPE intervention with concurrently assessing its actual implementation status. To the best of our knowledge, this is the first study to suggest the efficacy in of an IPE intervention on patient safety in promoting competency development among healthcare students in perinatal care.

Incorporating repeated assessments, our study showed significant improvements in self-reported IP competencies over time. These findings align with previous IPE evaluations using similar outcome measures [27, 31]. All three assessed competencies—IP collaboration, communication with patients, and with professionals—showed higher self-ratings at follow-up. Concurrently, perceptions of IPE relevance did not significantly increase, possibly due to ceiling or selection effects. Particularly, it was evident that relevance appraisals were particularly high at the baseline, with limited potential for increases over time. Furthermore, students with a high level of openness towards IP training may have subscribed or were selected based on personal engagements. However, participants’ interview statements corroborated individual gains in communication techniques, underscoring the feasibility of our IPE teaching format even among participants with potentially higher suitability at start.

While the three student groups reported similar competency levels at baseline, notable group differences occurred in observed changes over time; i.e., midwifery students showed most significant increases whereas medical student reported fewer change. These findings are consistent with previous IPE evaluation results, suggesting larger perceived benefits among non-medical participant groups [11]. However, observed group differences should be interpreted carefully and require further replication with larger study samples; also accounting for differences in patient safety knowledge prior to the intervention.

Both survey and interview findings revealed strong acceptance among project participants, underscoring program feasibility, along with a high value students attribute to interprofessional, practice-related learning opportunities [6, 11, 20, 23, 31, 33]. Project partners’ perspectives enriched the understanding of IPE implementation, reinforcing existing evidence on participants’ perceptions. Moreover, adding on findings regarding skill and behavioural development through IPE [10, 13], our results further contribute positive effects concerning encouragement of students to apply safety methods in clinical practice [20]. Interview findings emphasised that students’ sense of encouragement fostered their uptake of a disseminating role and peer knowledge sharing. This challenges previous claims that small-scale, pioneering IPE projects might have limited impact [16]. Additionally, further observations underscore the particular significance of combining technical and behavioural learning content in IPE [23, 32, 40].

To this end, our prospective, multi-method study addressed previously identified gaps in IPE evaluation [16, 18] by combining structured and competency-related assessment criteria with qualitative first-hand insights from student participants and project partners. Thus, our pilot findings contribute to the growing literature base on IPE approaches in health care and training programs as well as to the small literature base specifically in perinatal care.

### Limitations

Although we provide a novel IPE approach implemented in a real-world clinical setting with use of an elaborated, prospective evaluation design over an extended time, our results should be interpreted in the light of several limitations. We sought to establish a full sampling approach across the entire cohort of program participants and obtained a remarkably high participation rate. However, an overall limited sized sample was finally available for analysis. Our findings draw upon a pre-post design with a confined time lag and without a control group, which limits the ability to make causal inferences. In consideration of tailored, context- and content-specific criteria for evaluation, we utilised a self-designed questionnaire for self-report and established a prior consensus among researchers and practitioners. Nonetheless, we acknowledge that future studies should scrutinise further validity measures, reliabilities, as well psychometric properties of this item set (e.g., factorial validity). The development of additional, external measures for the assessment of IP competency levels among healthcare students has the potential to address this current shortcoming. Furthermore, our assessment relied on competency self-reports with no account taken of actual patient safety-related or clinical outcome measures.

This study should be conceived as a first pilot that needs to be followed-up by larger implementation trials across different educational and clinical settings. Subsequent attempts should incorporate more elaborated evaluation designs and should consider long-term outcomes, as well as patient-reported or patient safety outcomes [1, 13, 16, 18, 26, 30]. Furthermore, future evaluations should address inherent challenges to the implementation of IPE in routine clinical practice. These challenges include, but are not limited to, issues of consistent and long-term supervision, constrained resources in time and staff for teaching, as well as aligning curricula across different health professions’ study programs. Additionally, potential secondary benefits of IPE, such as long-term staff retention, may be considered for assessment of IPE implementation. Notwithstanding these constraining factors, our prospective results contribute to understanding of the potential competency trajectories of junior health professionals undergoing IPE training in patient safety.

## Conclusion

Our evaluation emphasises the potential of IPE interventions on patient safety for enhancing IP competency development in health professions’ education. Although this pilot implementation took place in a confined perinatal care setting, our findings may provide important insights for larger-scale studies that consider the issues of implementation and long-term sustainability. Although the informative value may be confined by small sample sizes and selection biases, we observed significant increments in competency levels over time, alongside consistently high levels of value placed on IPE by students. By addressing pertaining gaps in IPE evaluation through a multi-method and constructively aligned approach, this study further contributes first insights into implementation factors in a clinical practice setting. Our findings regarding effective content (i.e., communication techniques and perceived encouragement) and suitable didactic formats (i.e., small, interprofessional learning groups), may further provide a starting point for the design of future, larger-scale IPE approaches. Our results may further contribute to inform future investigations that seek to evaluate IPE interventions and associated learning, team, clinical, and patient-safety outcomes, also with regard to discussing potential limitations of evaluating practical training programs and IP competencies. Lastly, our results may help to justify further integration of IPE into health professions’ regular study curricula.

## Supplementary Information

Supplementary Material 1.

Supplementary Material 2.

Supplementary Material 3.

## Data Availability

The corresponding author will provide data upon reasonable request.

## Abbreviations

CNICF: Competency statement according to Canadian National Interprofessional Competency Framework
IP: Interprofessional
IPE: Interprofessional education
IPSTA: Interprofessional training ward (German: Interprofessionelle Ausbildungsstation)
ISBARR: Introduction–Situation–Background–Assessment–Recommendation–Read back
SiGerinn: Safety and Interprofessionality in Perinatal Care–Together and from the Beginning (German: Sicherheit und Interprofessionalität in der Geburtshilfe–Gemeinsam und von Beginn an)
P: Professional (for interview results)
S: Student (for interview results)
