# Supplementary material for: Implementation and effectiveness of an interprofessional educational intervention on patient safety in perinatal care: a multi-method, prospective evaluation study
Source: BMC Med Educ. 2026 Jul 9;26:1106. doi: 10.1186/s12909-026-09907-z (PMC13348609; doi:10.1186/s12909-026-09907-z)
Supplement: Supplementary file 1 — Supplementary Material 1. [file 12909_2026_9907_MOESM1_ESM.docx]

**APPENDIX I: Key competencies and learning objectives**

Table 3: Key competencies and learning objectives of the IPE intervention on patient safety

| **Key competency** | **Competency statement according to Canadian National Interprofessional Competency Framework (CNICF)** (32) | **Application - Aligned learning objectives for SiGerinn (Safety and Interprofessionality in Perinatal Care – Together and from the Beginning)** |
| --- | --- | --- |
| Interprofessional communication | ‘Learners/ practitioners from different professions communicate with each other in a collaborative, responsive and responsible manner.’ | - Students understand the interrelation of interprofessional communication and patient safety - Students can apply communication techniques (e.g. patient handover according to ISBARR*¹, Closed loop communication, Speak Up)*² - Students are confident to apply communication techniques |
| Patient-centredness | ‘Learners/ practitioners seek out, integrate and value, as a partner, the input and the engagement of the patient/client/ family/community in designing and implementing care/ services.’ | - Students understand the interrelatedness of patient-centredness and patient safety - Students understand the specific needs of patients in perinatal care - Students can actively communicate with patients and relatives, applying techniques such as ‘active listening’ and patient-centred values |
| Role clarification | ‘Learners/ practitioners understand their own role and the roles of those in other professions, and use this knowledge appropriately to establish and achieve patient/client/ family and community goals.’ | - Students understand the interrelatedness of role clarification and interprofessional collaboration as well as conflict resolution - Students understand roles and potential role conflicts in perinatal care - Students are able reflect their own and others’ professional roles |
| Interprofessional conflict resolution | ‘Learners/ practitioners actively engage self and others, including the client/patient/family, in positively and constructively addressing disagreements as they arise.’ | - Students understand the interrelatedness of interprofessional conflict resolution and patient safety - Students can anticipate interprofessional conflicts (situational awareness) - Students take an active role in preventing and solving interprofessional conflicts by applying communication techniques*² |

Notes: *¹ Patient handover according to ISBARR (Introduction–Situation–Background–Assessment– Recommendation–Read back), *² based on TeamSTEPPS (35).
