# Supplementary material for: Implementation and effectiveness of an interprofessional educational intervention on patient safety in perinatal care: a multi-method, prospective evaluation study
Source: BMC Med Educ. 2026 Jul 9;26:1106. doi: 10.1186/s12909-026-09907-z (PMC13348609; doi:10.1186/s12909-026-09907-z)
Supplement: Supplementary file 2 — Supplementary Material 2. [file 12909_2026_9907_MOESM2_ESM.docx]

**APPENDIX II: Survey instruments**

Table 4: Categories and questionnaire items of the survey on interprofessional competency evaluation (for baseline and follow-up survey)

| **SURVEY QUESTIONNAIRE** | |
| --- | --- |
| **Category 1 - Interprofessional collaboration** | |
| 1.1 | At the start of the assignment, I communicate my current skill level to the team. |
| 1.2 | I am familiar with my team members’ competencies. |
| 1.3 | I communicate my daily task with my team. |
| 1.4 | My colleagues and I actively coordinate the next steps of our work. |
| 1.5 | I actively offer mutual support to my colleagues. |
| 1.6 | If a situation is unclear to me, I reach out to my direct supervisor. |
| 1.7 | I have a clear understanding of my team members’ professional roles. |
| **Category 2 – Communication with patients and relatives** | |
| 2.1 | I respect the personal boundaries of patients and their relatives |
| 2.2 | I introduce myself to the patients and their relatives, including my current level of studies. |
| 2.3 | I plan the discharge together with the patients and their relatives. |
| 2.4 | I am able to correctly identify patients. |
| 2.5 | I encourage patients and their relatives to use Speak Up. |
| 2.6 | I encourage patients and their relatives to actively participate in their treatment. |
| **Category 3 – Interprofessional communication techniques** | |
| 3.1 | I can correctly perform a structured patient handover (e.g. according to ISBARR) |
| 3.2 | I ask directly if I do not understand something in direct interprofessional collaboration. |
| 3.3 | I apply Speak Up with my colleagues. |
| 3.4 | I encourage my colleagues to use Speak Up. |
| 3.5 | In critical situations, I actively listen to my colleagues. I repeat aloud what I have heart and what has been done. |
| 3.6 | In an unclear situation, I conduct a quick alignment/ briefing with my colleagues. |
| 3.7 | After we have managed something unexpected, I conduct a brief feedback/ debriefing with my colleagues. |
| 3.8 | In an emergency situation, I apply Closed loop communication. |
| 3.9 | In an unclear situation, I apply the ’10 seconds for 10 minutes’ communication technique. |
| **Category 4 – Perceptions of interprofessional education** | |
| 4.1 | Learning together will help me think positively about other professions. |
| 4.2 | Learning with students from other healthcare professions enhances my teamwork skills. |
| 4.3 | Interprofessional education during my studies will help me work better in a team. |

Notes on Category 4: Items were taken from the *German Interprofessional Attitude Scale* (TRR1, TRR2, TRR3) by Pedersen et al. (published under the Creative Commons Attribution 4.0 International License, CC BY 4.0) (41), and translated from German to English for the present publication.

Table 5: Interview guide (main questions) for semi structured in-depth interviews for evaluation of project implementation

| **INTERVIEW GUIDE (MAIN QUESTIONS)** | | | |
| --- | --- | --- | --- |
| **Outcome** | **Definition** (Proctor et al. (42)*) | **Interview questions** | |
|  |  | Project partners (professionals) | Project participants (students) |
| Acceptability | ‘the perception among implementation stakeholders that a given treatment, service, practice, or innovation is agreeable, palatable, or satisfactory’ | How did you like contents and delivery of the program? | |
| Adoption | ‘the intention, initial decision, or action to try or employ an innovation or evidence-based practice’ | At the beginning, what did you/ other stakeholders think about the project idea? | - |
| Appropriateness | ’perceived fit, relevance, or compatibility of the innovation or evidence based practice for a given practice setting, provider, or consumer; and/or perceived fit of the innovation to address a particular issue or problem’ | How relevant was the program for you/ participants? | |
| Feasibility | ’the extent to which a new treatment, or an innovation, can be successfully used or carried out within a given agency or setting’ | What do you think about the format, i.e. the combined implementation of the interprofessional training ward and SiGerinn? | |
| Fidelity | ’as the degree to which an intervention was implemented as it was prescribed in the original protocol or as it was intended by the program developers’ | Our objective was to develop patient safety training, focusing on interprofessional collaboration and communication. We aimed to teach methodical competencies (communication techniques) and self-confidence to apply patient safety behaviour in care practice. Do you think we met this aim? Why/ Why not? | |
| Penetration | ’the integration of a practice within a service setting and its subsystems’ | How do you perceive the reach of our project? | |
| Sustainability | ’the extent to which a newly implemented treatment is maintained or institutionalized within a service setting’s ongoing, stable operations’ | Will the project further exist? Why/ Why not? | - |
|  |  | - | How do you perceive the long-term benefits of the program? |

Notes: Translated; interviews were conducted in German; *This evaluation focused on processual and content-related outcomes and did not include economic outcomes. Therefore, the outcome ‘implementation costs’ (42) has not been adopted.
