# Supplementary material for: Implementation and effectiveness of an interprofessional educational intervention on patient safety in perinatal care: a multi-method, prospective evaluation study
Source: BMC Med Educ. 2026 Jul 9;26:1106. doi: 10.1186/s12909-026-09907-z (PMC13348609; doi:10.1186/s12909-026-09907-z)
Supplement: Supplementary file 3 — Supplementary Material 3. [file 12909_2026_9907_MOESM3_ESM.docx]

**APPENDIX III: Complementary result data**

Table 6: Results of the survey items in baseline and follow-up with statistical comparison

|  |  | **Pre-Post Evaluation** | | **Test for difference** | | |
| --- | --- | --- | --- | --- | --- | --- |
| **Survey category** | **Survey item** | **Baseline** | **Follow-up** |  |  |  |
|  |  | M (SD) | M (SD) | t | p | g |
| Interprofessional collaboration | 1.1 | 4.20 (1.06) | 5.05 (1.10) | 2.82 | 0.011 | 0.61 |
|  | 1.2 | 3.60 (1.05) | 4.85 (0.99) | 4.32 | **<0.001** | **0.93** |
|  | 1.3 | 4.30 (1.26) | 5.50 (0.61) | 4.86 | **<0.001** | **1.04** |
|  | 1.4 | 4.20 (0.95) | 5.60 (0.60) | 8.30 | **<0.001** | **1.78** |
|  | 1.5 | 5.30 (0.73) | 5.55 (0.69) | 1.42 | 0.171 | 0.31 |
|  | 1.6 | 5.15 (0.88) | 5.45 (0.76) | 1.67 | 0.110 | 0.36 |
|  | 1.7 | 3.85 (0.75) | 5.15 (0.81) | 6.30 | **<0.001** | **1.35** |
| Communication with patients and relatives | 2.1 | 5.05 (0.83) | 5.40 (0.60) | 1.68 | 0.110 | 0.36 |
|  | 2.2 | 4.80 (1.11) | 5.05 (1.61) | 0.72 | 0.480 | 0.16 |
|  | 2.3 | 3.05 (1.19) | 4.70 (1.30) | 6.77 | **<0.001** | **1.45** |
|  | 2.4 | 3.95 (1.47) | 4.95 (1.70) | 2.24 | 0.038 | 0.48 |
|  | 2.5 | 2.35 (1.27) | 3.50 (1.28) | 3.29 | **0.004** | 0.71 |
|  | 2.6 | 3.50 (1.24) | 4.85 (1.09) | 4.61 | **<0.001** | **0.99** |
| Interprofessional communication techniques | 3.1 | 4.00 (0.97) | 5.00 (0.97) | 3.16 | 0.005 | 0.68 |
|  | 3.2 | 4.85 (1.09) | 5.25 (0.97) | 1.36 | 0.189 | 0.29 |
|  | 3.3 | 2.85 (1.50) | 4.25 (1.25) | 3.99 | **<0.001** | **0.86** |
|  | 3.4 | 2.30 (1.22) | 3.90 (1.21) | 5.29 | **<0.001** | **1.14** |
|  | 3.5 | 3.30 (1.08) | 4.65 (1.89) | 8.10 | **<0.001** | **1.74** |
|  | 3.6 | 4.05 (1.27) | 5.50 (0.61) | 4.78 | **<0.001** | **1.03** |
|  | 3.7 | 4.00 (1.17) | 5.10 (1.07) | 3.49 | **0.002** | 0.75 |
|  | 3.8 | 2.75 (1.25) | 4.20 (1.40) | 3.45 | **0.003** | 0.74 |
|  | 3.9 | 1.80 (0.95) | 3.45 (1.61) | 3.94 | **<0.001** | **0.85** |
| Perceptions of interprofessional education | 4.1 | 4.95 (0.22) | 4.95 (0.22) | 0.00 | 1.000 | 0.00 |
|  | 4.2 | 4.95 (0.22) | 4.90 (0.31) | 1.00 | 0.330 | 0.22 |
|  | 4.3 | 4.95 (0.22) | 4.90 (0.31) | 1.00 | 0.330 | 0.22 |

Notes: n=20 participants, M­–Mean, SD–Standard deviation, p–Significance level, bold if p (adjusted) ≤ 0.004, g–Effect size, bold if Hedges’ g ≥ 0.8.
